# Excitation of prefrontal cortical neurons during conditioning enhances fear memory formation

**DOI:** 10.1038/s41598-020-65597-7

**Published:** 2020-05-25

**Authors:** Natsumi Shibano, Mio Yamazaki, Tomoki Arima, Konami Abe, Marin Kuroda, Yuki Kobayashi, Shigeyoshi Itohara, Teiichi Furuichi, Yoshitake Sano

**Affiliations:** 1 Department of Applied Biological Science, Tokyo University of Science, Noda Chiba, 278-8510 Japan; 2 Laboratory for Behavioral Genetics, RIKEN Brain Science Institute, Wako Saitama, 351-0198 Japan; 3 Present Address: BRIN/MINDS, RIKEN Center for Brain Science, Wako, Saitama 351-0198 Japan

**Keywords:** Cognitive neuroscience, Emotion, Learning and memory, Fear conditioning

## Abstract

Animals can remember a situation associated with an aversive event. Contextual fear memory is initially encoded and consolidated in the hippocampus and gradually consolidated in multiple brain regions over time, including the medial prefrontal cortex (PFC). However, it is not fully understood how PFC neurons contribute to contextual fear memory formation during learning. In the present study, neuronal activity was increased in PFC neurons utilizing the pharmacogenetic hM3Dq-system in male mice. We show that fear expression and memory formation are enhanced by increasing neuronal activity in PFC during conditioning phase. Previous studies showed that the activation of hM3Dq receptor in a subset of amygdala neurons enhanced fear memory formation and biased which neurons are allocated to a memory trace, in which immediate early gene c-fos was preferentially expressed following memory retrieval in these pre-activated neurons. In this study, hM3Dq activation in PFC could not change the probability of c-fos expression in pre-activated neurons flowing memory retrieval. Instead, the number c-fos positive neurons following memory retrieval was significantly increased in the basolateral amygdala. Our results suggest that neuronal activity in PFC at the time of learning modulates fear memory formation and downstream cellular activity at an early phase.

## Introduction

Memories of fearful events can be retained for extended periods of time, which allows animals to predict danger; thus, this process is important for their survival. In humans, the formation of strong fear memory can drive the development of neuropsychiatric disorders, such as posttraumatic stress disorder (PTSD). Both the hippocampus and amygdala are critical structures for contextual fear memory formation^1,2^. The contribution of the hippocampus to contextual fear memory retrieval decreases over time^1,3,4^. Alternatively, some cortical areas such as the PFC and the anterior cingulate cortex gradually become more impactful on memory retrieval^5–8^. It is reported that PFC activity is associated with inappropriate fear in PTSD patients^9^. The dorsal and ventral subdivisions of PFC regulate the expression and extinction of fear in rodents, respectively^10^. Inactivation of the dorsal PFC interferes with the expression of fear in cued and contextual fear conditioning (CFC)^11–15^. In the PFC, expression of conditioned fear is temporally controlled by oscillation of functional assemblies^16^, and stimulation of the BLA to PFC projection increases cue-associated freezing behavior^17^. In contrast, inactivation of PFC and stimulating PFC to BLA projection during conditioning has a small influence on memory consolidation at an early phase^11,18^. Nonetheless, it has been shown that micro-stimulation of the PFC during tone representation increases conditioned fear expression under low foot shock conditions^19^. However, the contribution of PFC to consolidation in CFC at an early phase of learning is not fully understood.

Recent works show that a subset of PFC neurons is rapidly tagged during CFC and memory is gradually consolidated in those neurons, called engram cells^7,8^. The tagged PFC neurons do not contribute to the retrieval of contextual fear memory in a day after learning and can contribute to physiological memory retrieval up to several weeks later^7,8^. Previous studies have shown that a subset of neurons activated during learning was preferentially incorporated into a given memory trace, turning into engram cells^20,21^. We and other researchers suggest that this recruitment (i.e., memory allocation) is regulated by neural excitability and/or activity of the cAMP response element-binding protein (CREB) in the hippocampus, amygdala and cortex^22–29^. In a previous study^26^, the excitation of a random small population of amygdala neurons using hM3Dq with CS (conditioned stimulus) - US (unconditioned stimulus) association enhanced memory formation during cued fear conditioning and biased which neurons are involved in the fear memory retrieval.

In this study, we manipulated neuronal activity during the conditioning of a subset of PFC neurons expressing hM3Dq receptors. Next, we tested whether the activation of PFC neurons regulated fear memory formation in the CFC and analyzed whether hM3Dq activation in PFC neurons during conditioning modulated the process of memory allocation during early learning phase, based on the expression of the c-fos protein. We also identified the brain areas that are modulated by PFC stimulation in the CFC.

## Results

### Increasing neuronal activity in PFC neurons utilizing hM3Dq systems during conditioning enhanced the expression of fear and memory formation

**Figure 1:**
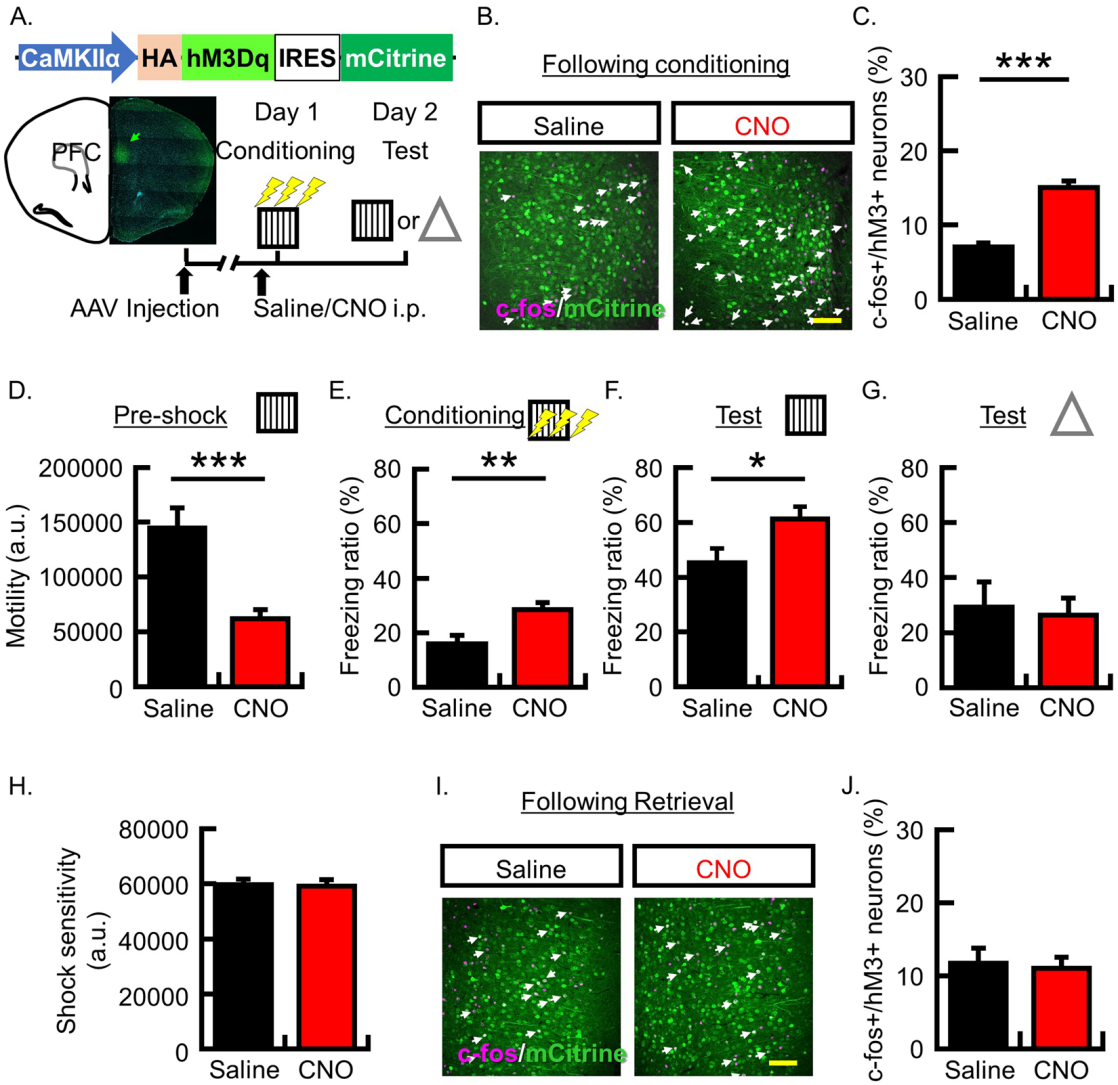
Effect of increasing neuronal activity during contextual fear conditioning by stimulating hM3Dq expressed in PFC neurons. (**A**) The top panel shows the map of the AAV construct. Bottom panel shows experimental schema. Representative picture showing localized mCitrine (green) expression in PFC by hM3Dq-IRES-mCitrine virus infection. (**B**) Representative images showing expression of mCitrine (green) and c-fos (magenta) following conditioning. (**C**) Probability of cells expressing c-fos following the conditioning in hM3Dq+ neurons (N = 4 animals per group). (**D**) Data show the mean locomotor activity for 3 min during pre-shock periods. (**E**) Data show the mean freezing ratio for 3 min during the shock-presenting period in the conditioning session. (**F**) Data show the mean freezing ratio for 5 min during the retrieval test in the conditioned context (D-F, saline group, N = 13; CNO group, N = 12 animals). (**G**) Data show the mean freezing ratio for 5 min during the retrieval test in the novel context (N = 5 animals in each group). (**H**) Data show the shock sensitivity (saline group, N = 13; CNO group, N = 12 animals). (**I**) Representative images showing expression of mCitrine (green) and c-fos (magenta) following retrieval in the conditioned context. (**J**) Probability of cells expressing c-fos following the conditioning in hM3Dq+ neurons (saline group, N = 6; CNO group, N = 7 animals). Scale bars = 100 um. White arrows indicate double-labeled neurons (c-fos+ and hM3Dq+). Data are represented as means ± SEM; *p < 0.05, **p < 0.01, and ***p < 0.001 (saline-injected mice, black columns; CNO-injected mice, red columns).

We used the modified human muscarinic acetylcholine receptor type 3 (hM3Dq) to manipulate neuronal activity. The hM3Dq is Gq protein coupled receptor and has no constitutive activity to increase neuronal activity^30^. The synthetic ligand clozapine-N-oxide (CNO) but not endogenous ligands for muscarinic receptors can selectively bind to and activate the hM3Dq receptors, which result in increased membrane potential of approximately 5–10 mV^26,30^. Here, hM3Dq was expressed in PFC to test whether the activation of PFC neurons by hM3Dq stimulation during learning modulates fear memory formation (Fig. 1A). To express hM3Dq in PFC neurons, CaMKIIa-HA-hM3D(Gq)-IRES-mCtrine (10^12^ gc/mL) adeno-associated virus (AAV) was infused bilaterally into the PFC according to the brain atlas^31^. Immunohistochemical studies with an antibody against GFP detected the expression of this viral gene in a region approximately 1 mm away from the injection sites in the PFC (Supplementary Fig. 1). In this experiment, the hM3Dq receptor was expressed in 40.3% ± 2.6% of neurons around the injection site. These mice were then subjected to CFC (Fig. 1A). To increase neuronal activity in PFC neurons, 3 mg/kg CNO was systemically administrated 30 min before training. A memory retrieval test was implemented 1 day following the CFC in the absence of CNO.

First, we analyzed whether hM3Dq-positive neurons are preferentially activated following conditioning by administration of CNO. To visualize these neurons, we used the c-fos protein. The expression of c-fos is rapidly and transiently induced by neuronal activity with the elevation of intracellular Ca^2+^ concentration. The expression of c-fos protein in hM3Dq-positive neurons was significantly higher in the CNO group than in the saline group (Fig. 1B,C; c-fos+/hM3Dq+ neurons (%), N = 4 in each group: saline group, 7.1 ± 0.5; CNO group, 15.0 ± 0.9; p = 0.0003). The c-fos expression was significantly higher in the hM3Dq positive cells compared with negative neighbor cells in the CNO group but not in the saline group (N = 4 in each group: CNO group, hM3Dq+/c-fos+ cells (%), 61.6 ± 3.2; hM3Dq-/c-fos+ cells (%), 38.4 ± 3.2; p = 0.036; saline group, hM3Dq+/c-fos+ cells (%), 53.0 ± 4.4; hM3Dq-/c-fos+ cells (%), 47.0 ± 4.4; p = 0.55). These results indicate that neuronal activation is preferentially induced in hM3Dq-positive neurons by CNO administration. Next, we tested whether PFC stimulation modulates locomotor activity and fear expression during conditioning. The locomotor activity in the context before presenting shocks was significantly decreased in the CNO group compared with the saline group (Fig. 1D; motility (a.u.): saline group, 144,875 ± 18,390, N = 13; CNO group, 61,896 ± 8,446, N = 12; p = 0.0006). Freezing behavior during the shock-presenting period was significantly higher in the CNO group than in the saline group (Fig. 1E; freezing ratio (%): saline group, 16.0 ± 3.0, N = 13; CNO group, 28.5 ± 2.5, N = 12; p = 0.005). However, shock sensitivity was not significantly different between the saline and CNO groups (Fig. 1H; shock sensitivity (a.u.): saline group, 59,680 ± 2,052, N = 13; CNO group, 59,109 ± 2,386, N = 12; p = 0.86).

Next, a memory retrieval test was implemented one day following the CFC in the absence of CNO. Freezing behavior was significantly higher in the CNO group than in the saline group (Fig. 1F; freezing ratio (%): saline group, 45.3 ± 5.1, N = 13; CNO group, 61.2 ± 4.6, N = 12; p = 0.03). Furthermore, we addressed whether PFC activation using hM3Dq non-specifically enhances fear expression. Following our methodology, CNO was systemically administrated 30 min before training. Then, the mice were tested in a novel context, which is different from the conditioned context. In this retrieval test, the freezing behavior was not significantly different between the two groups (Fig. 1G; freezing ratio (%): N = 5 in each group, saline group, 29.3 ± 9.0; CNO group, 26.4 ± 6.3; p = 0.80). These results suggest that hM3Dq activation in PFC neurons during conditioning enhances the fear memory formation at an early phase.

A previous study has shown that hM3Dq activation in a subset of amygdala neurons enhances memory formation in the auditory fear conditioning and biased which neurons are incorporated into a fear memory trace^26^. Then, we analyzed whether pre-activated PFC neurons were preferentially activated by memory retrieval. Next, we imaged c-fos protein following the memory retrieval test and analyzed the colocalization of c-fos protein in hM3Dq-positive neurons. No difference was found between the CNO and saline group for the probability of c-fos expression in hM3Dq-positive neurons (Fig. 1I,J; c-fos+/hM3Dq+ neurons (%): saline group, 11.8 ± 2.1, N = 6; CNO group, 11.0 ± 1.5, N = 7; p = 0.78). These data suggest that hM3Dq activation in PFC neurons do not bias which neurons are reactivated during memory retrieval at an early phase.

**Figure 2:**
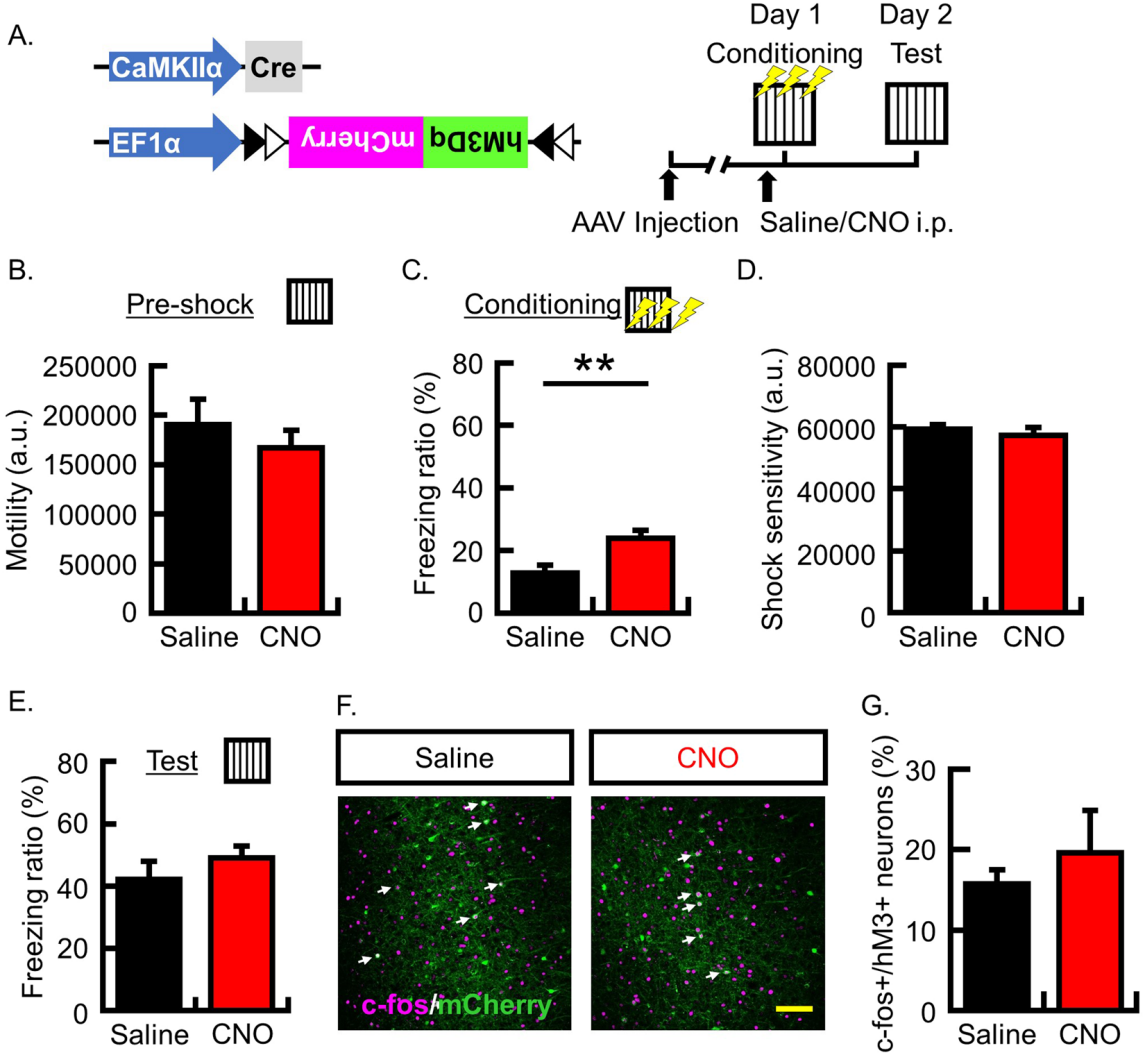
Manipulation of neuronal activity in a small subset of PFC neurons. (**A**) The left panel shows the map of the AAV constructs; the right panel shows the experimental schema. (**B**) Mean locomotor activity for 3 min during the pre-shock period. (**C**) Mean freezing ratio for 3 min during the shock-presenting period of the conditioning session. (**D**) Shock sensitivity. (**E**) Mean freezing ratio for 5 min during the retrieval test in the conditioned context (B-E, N = 17 animals in each group). (**F**) Representative images showing expression of mCherry (green) and c-fos (magenta) after retrieval. (**G**) Probability of cells expressing c-fos following conditioning in hM3Dq+ neurons (N = 6 animals in each group). Scale bars = 100 μm. White arrows indicate double-labeled neurons (c-fos+ and hM3Dq+). Data are represented as means ± SEM; **p < 0.01 (saline-injected mice, black columns; CNO-injected mice, red columns).

In past research, neuronal activity was manipulated in a small random population (~10%) of neurons^26,28^ to induce neuronal competition. Then, to express hM3Dq in a small subset of PFC neurons, we utilized the Cre-dependent hM3Dq expression system: mixing EF1a-DIO-hM3Dq and diluted CaMKIIa-Cre (10^9^ gc/mL) AAVs and infusing the solution bilaterally into the PFC (Fig. 2A). In this experiment, the hM3Dq receptor was expressed in 7.6% ± 0.7% of neurons around the injection site. Mice were then subjected to CFC. Following our methodology, CNO was systemically administrated 30 min before training and a memory retrieval test was performed 1 day following CFC. In the manipulation of a small subset of neurons, the locomotor activity during pre-shock periods was not significantly different between the saline and CNO groups (Fig. 2B; motility (a.u.): N = 17 in each group, saline group, 190,332 ± 25,723; CNO group, 166,851 ± 17,758; p = 0.46). However, freezing behavior during the shock-presenting period was significantly higher in the CNO group than in the saline group (Fig. 2C; freezing ratio (%): N = 17 in each group, saline group, 12.9 ± 2.4; CNO group, 23.9 ± 2.5; p = 0.004). Similarly, shock sensitivity showed no significant difference between the saline and CNO groups (Fig. 2D; shock sensitivity (a.u.): N = 17 in each group, saline group, 59,251 ± 1,460; CNO group, 57,318 ± 2,564; p = 0.52). In the retrieval test, the freezing ratio was not significantly different between the saline and CNO groups (Fig. 2E; freezing ratio (%): N = 17 in each group, saline group, 42.2 ± 5.7; CNO group, 49.1 ± 3.8; p = 0.32). Our analysis of the colocalization of c-fos protein in hM3Dq-positive neurons revealed that there was no significant difference between the CNO and saline groups (Fig. 2F,G; c-fos+/hM3Dq+ neurons (%), N = 6 in each group: saline group, 15.7 ± 1.8; CNO group, 19.6 ± 5.3; p = 0.50).

Considering the above results, our study suggests that increased PFC activity enhances fear expression during conditioning and memory formation. Although the manipulation of neuronal activity in PFC neurons using hM3Dq systems did not induce a change in which neurons were preferentially activated following recent memory retrieval, our results do not conclusively reject the possibility that memory allocation in PFC is regulated by neuronal activity during the learning phase. To elucidate this potential effect, future studies should investigate specific manipulations of the neuronal circuit and analyze remote memory retrieval.

### Lateral hypothalamus neurons were activated following conditioning by activation of hM3Dq expressed in PFC neurons

**Figure 3:**
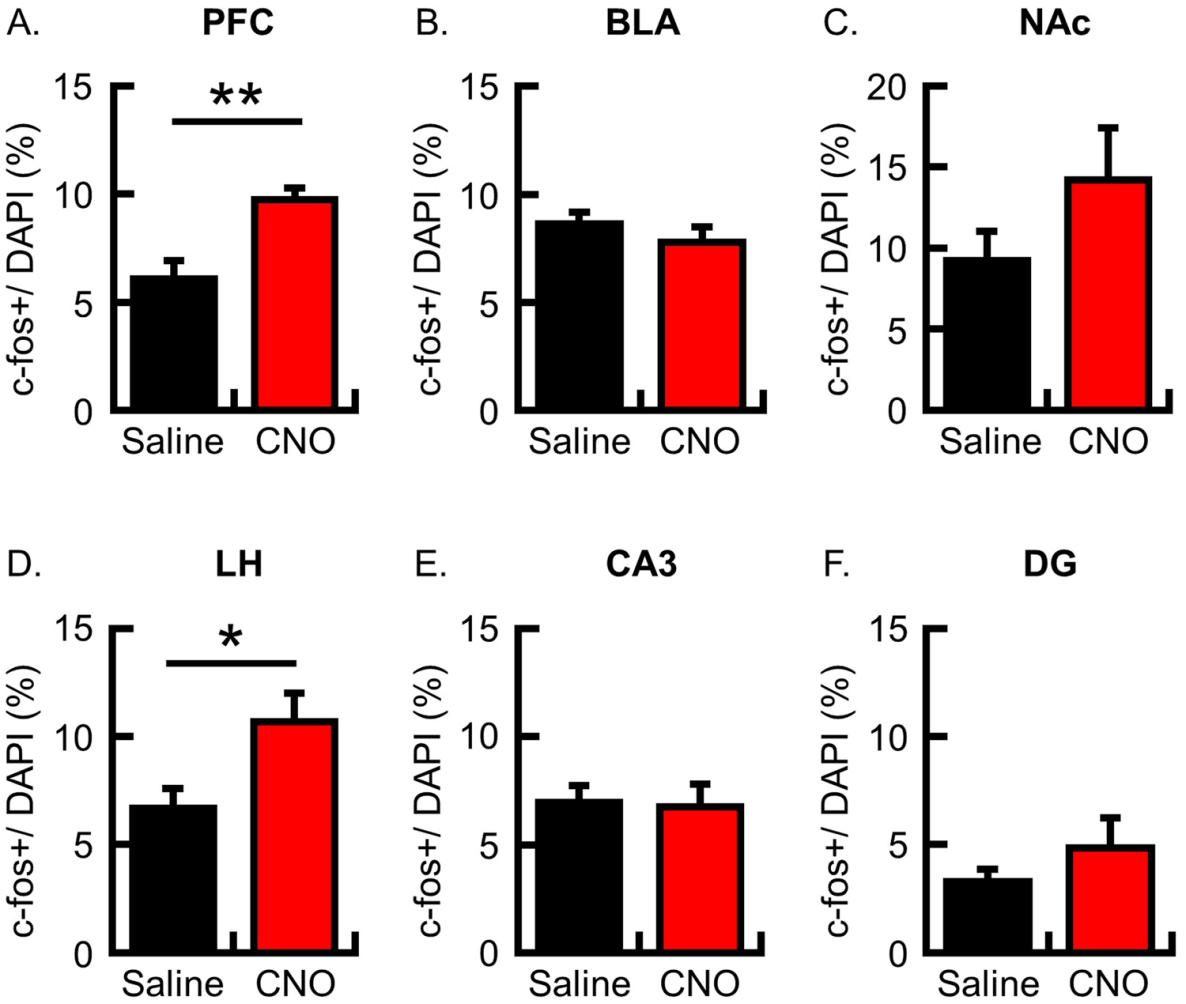
Analysis of c-fos-expressing neurons following conditioning with activation of hM3Dq expressed in PFC neurons. The c-fos protein was imaged flowing conditioning in the brain infused CaMKIIa-hM3Dq-IRES-mCitrine AAV. (**A–F**) The probability of cells expressing c-fos following fear conditioning was analyzed in the PFC (**A**), BLA (**B**), NAc (**C**), LH (**D**), CA3 (**E**) and DG (**F**) (N = 6 animals per group). The number of c-fos+ neurons is normalized by the number of DAPI + cells in the analyzed area. Data are represented as means ± SEM; *p < 0.05 and **p < 0.01 (saline-injected mice, black columns; CNO-injected mice, red columns).

Freezing behavior during conditioning was increased by activation of hM3Dq expressed in PFC neurons. To identify brain areas modulated by PFC activation, we analyzed the expression of c-fos following CFC in some brain areas, in which mice CaMKIIa-hM3Dq AAV was infused into PFC. In the PFC where hM3Dq was expressed, the number of c-fos-positive neurons was significantly increased in the CNO group compared with the saline group (Fig. 3A; c-fos+/DAPI (%), N = 6 in each group: saline group, 6.1 ± 0.9; CNO group, 9.7 ± 0.5, p = 0.005), which is consistent with increased neuronal activity using hM3Dq. PFC neurons innervate the BLA, nucleus accumbens (NAc) and lateral hypothalamus (LH). BLA is reciprocally connected with PFC, which circuits regulate fear expression^14,17^. Contrary to our prediction, the number of c-fos positive neurons was no difference between saline and CNO group in the BLA (Fig. 3B; c-fos+/DAPI (%), N = 6 in each group: saline group, 8.6 ± 0.5; CNO group, 7.8 ± 0.7; p = 0.36). In the NAc, there were no differences between the saline and CNO group (Fig. 3C; c-fos+/DAPI (%), N = 6 in each group; NAc, saline group, 9.2 ± 1.8; CNO group, 14.2 ± 3.2; p = 0.20). PFC and BLA innervate the LH^32^ which is involved in fear memory learning^33–35^. In the LH, the number of c-fos positive neurons was significantly increased in the CNO group compared with saline group (Fig. 3D; c-fos+/DAPI (%), N = 6 in each group: saline group, 6.7 ± 0.9; CNO group, 10.7 ± 1.3; p = 0.03). We also analyzed hippocampal areas that are critical for CFC. In the CA3 and dentate gyrus (DG), the number of c-fos positive neurons was indistinguishable between the two groups (Fig. 3E,F; c-fos+/DAPI (%), N = 6 in each group: CA3, saline group, 7.0 ± 0.8, CNO group, 6.8 ± 1.1, p = 0.88; DG, saline group, 3.3 ± 0.6, CNO group, 4.8 ± 1.4, p = 0.32).

**Figure 4:**
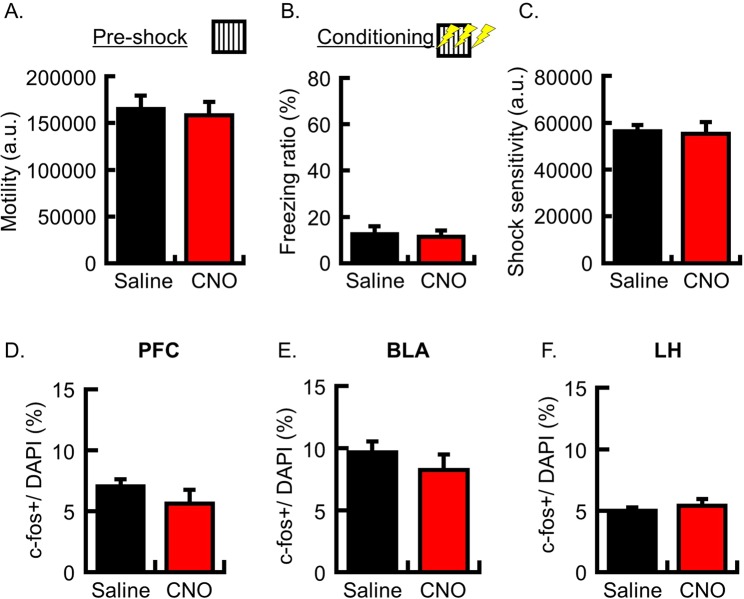
Analysis of c-fos-expressing neurons following conditioning in naïve mice. (**A**) Mean locomotor activity for 3 min during the pre-shock period. (**B**) Mean freezing ratio for 3 min during the shock-presenting period of the conditioning session. (**C**) Shock sensitivity (**A–C**; saline group, N = 8; CNO group, N = 7 animals). (**D–F**) Probability of cells expressing c-fos following fear conditioning, as analyzed in the PFC (**D**), BLA (**E**) and LH (**F**) (N = 5 animals per group). The number of c-fos+ neurons is normalized by the number of DAPI + cells in the analyzed area. Data are represented as means ± SEM (saline-injected mice, black columns; CNO-injected mice, red columns).

To exclude the possibility that increased c-fos expression in PFC and LH was caused by CNO administration, we analyzed c-fos expression in naïve mice with no hM3Dq transduced. Mice were then subjected to CFC; either CNO or saline was systemically administrated 30 min before conditioning, and the mice were perfused for brain sampling 90 min after conditioning. The locomotor activity during the pre-shock period (Fig. 4A; motility (a.u.); saline group, 165,082 ± 14,250, N = 8; CNO group, 158,469 ± 14,294, N = 7; p = 0.75), the freezing ratio during the shock-presenting period (Fig. 4B; freezing ratio (%); saline group, 12.4 ± 3.5, N = 8; CNO group, 11.5 ± 2.7, N = 7; p = 0.84), and shock sensitivity (Fig. 4C; shock sensitivity (a.u.); saline group, 56,391 ± 2,703, N = 8; CNO group, 55,285 ± 4,988, N = 7; p = 0.84) were not significantly different between the saline and CNO groups. These results support the prediction that increased freezing behavior during the shock-representing period (shown in Figs. 1E and 2C) is caused by the stimulation of hM3Dq in the PFC. The c-fos imaging analysis showed that the number of c-fos positive neurons was not significantly different between the saline and CNO groups in the PFC, BLA and LH (Fig. 4D–F; c-fos+/DAPI (%), N = 5 in each group: PFC, saline group, 7.0 ± 0.6, CNO group, 5.6 ± 1.1, p = 0.30; BLA, saline group, 9.7 ± 0.9, CNO group, 8.2 ± 1.3, p = 0.38; LH, saline group, 5.0 ± 0.3, CNO group, 5.4 ± 0.5, p = 0.54). These results suggest that c-fos expression in LH (Fig. 3D) is increased by the stimulation of hM3Dq expressed in a subset of PFC neurons during conditioning.

### BLA neurons were more activated following memory retrieval in PFC stimulated mice

**Figure 5:**
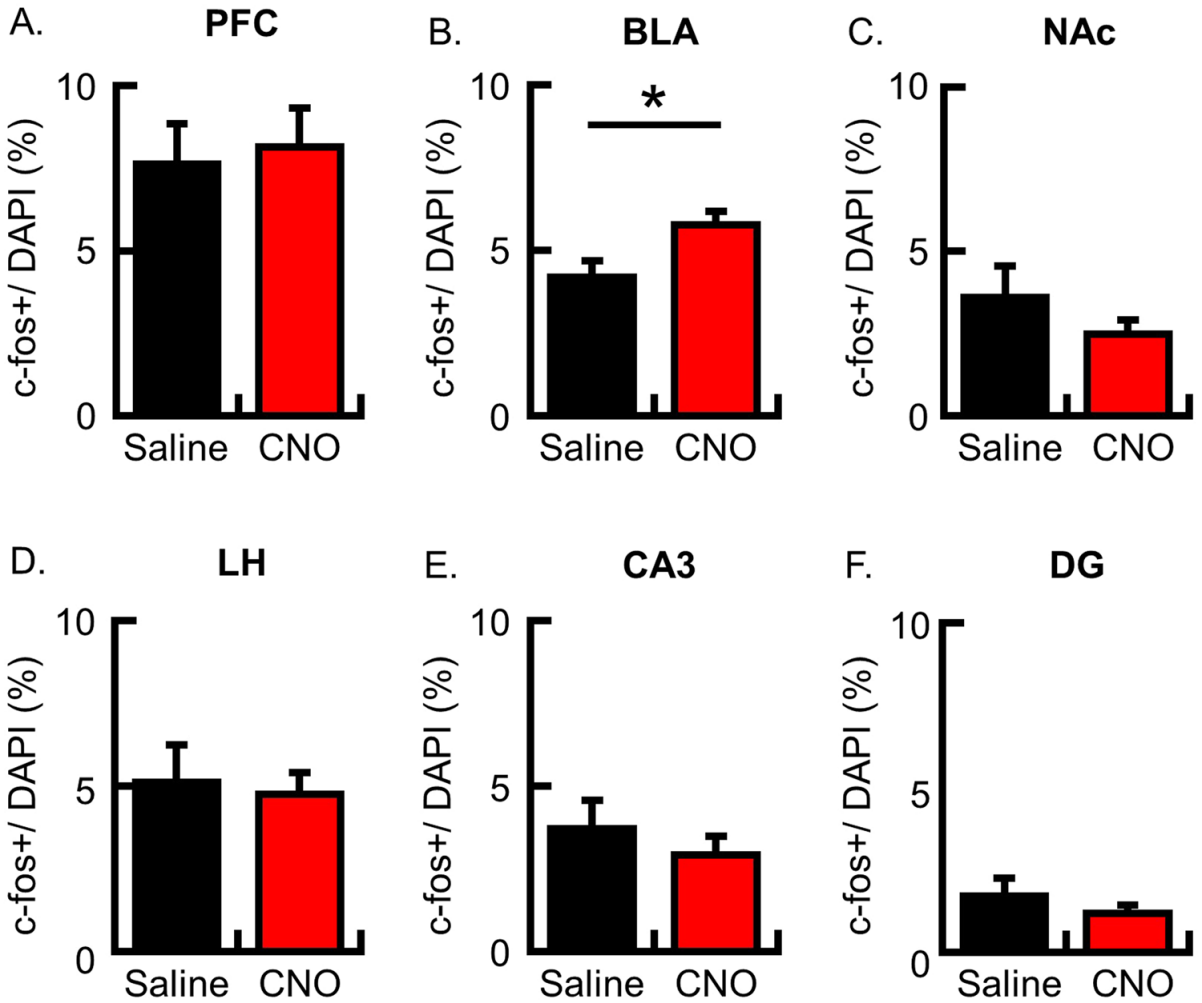
Analysis of c-fos-expressing neurons following memory retrieval. The c-fos protein was imaged flowing retrieval in the brain infused CaMKIIa-hM3Dq-IRES-mCitrine AAV. (**A–F**) The probability of cells expressing c-fos following memory retrieval was analyzed in the PFC (**A**), BLA (**B**), NAc (**C**), LH (**D**), CA3 (**E**) and DG (**F**) (A, saline group, N = 6; CNO group, N = 7 animals; B–F, N = 6 animals per group). The number of c-fos+ neurons is normalized by the number of DAPI + cells in the analyzed area. Data are represented as means ± SEM; *p < 0.05 (saline-injected mice, black columns; CNO-injected mice, red columns).

Fear memory formation was enhanced by stimulating PFC neurons using hM3Dq during conditioning. Next, we analyze the c-fos expression following memory retrieval. In the PFC, the number of c-fos-positive neurons after the memory retrieval test was not significantly difference between the saline and CNO group (Fig. 5A; c-fos+/DAPI (%): saline group, 7.6 ± 1.2, N = 6; CNO group, 8.1 ± 1.2, N = 7; p = 0.76). This result would support the idea that the increased freezing behavior in the CNO group was not caused by facilitation of memory recruitment into the PFC (Fig. 1J). Interestingly, the number of c-fos-positive neurons in the BLA was significantly increased in the CNO group compared with the saline group (Fig. 5B; c-fos+/DAPI (%), N = 6 in each group: saline group, 4.2 ± 0.5; CNO group, 5.8 ± 0.4; p = 0.03). In contrast, there were no differences between the two groups in other brain areas (Fig. 5C–F; c-fos+/DAPI (%), N = 6 in each group: NAc, saline group, 3.6 ± 1.0, CNO group, 2.5 ± 0.4, p = 0.32; LH, saline group, 5.1 ± 1.1, CNO group, 4.7 ± 0.7, p = 0.78; CA3, saline group, 3.7 ± 0.9, CNO group, 2.9 ± 0.6, p = 0.46; DG, saline group, 1.7 ± 0.5, CNO group, 1.2 ± 0.3, p = 0.39). Taken together, these results possibly suggest that PFC activation during conditioning would activate downstream circuits including the LH and BLA, facilitate CS-US association in the BLA (though activation in BLA neurons during conditioning was not detected in the c-fos imaging analysis) and more BLA neurons could be activated by CS representation in retrieval test (Supplementary Fig. 2).

### Fear expression is augmented by increasing noradrenergic activity in the PFC

Freezing behavior during conditioning was enhanced by stimulating PFC neurons, and fear memory formation was enhanced. PFC neurons project to the LH^32^ which innervate noradrenergic neuron in the locus coeruleus (LC)^33,34^. The stimulation of LH to LC pathway enhances fear memory formation^33^. Noradrenergic neurons in the LC are activated under stressful conditions and modulates fear memory formation^36^. LC triggers relapse by altering PFC firing dynamics to drive fear expression^37^. Then, we tested whether activation of adrenergic receptor in PFC enhances fear expression. In this experiment, we used a phenylephrine, an α1 adrenergic receptor (α1-AR) agonist. The α1-AR is a Gq protein-coupled receptor, which is implicated in the functions of the PFC, such as emotion, stress response and cognition^38–41^. Phenylephrine was infused into the PFC via a cannula before CFC (Supplementary Fig. 3A). The locomotor activity during pre-shock period was significantly decreased in the phenylephrine group compared with the saline group (Supplementary Fig. 3B, motility (a.u.); N = 15 in each group: saline group, 52,261 ± 8,017; phenylephrine group, 24,936 ± 7,809; p = 0.02). Freezing ratio during the shock-presenting period was significantly higher in the phenylephrine group compared with the saline group (Supplementary Fig. 3 C, freezing ratio (%), N = 15 in each group: saline group, 30.9 ± 5.2; CNO group, 55.1 ± 4.9; p = 0.002). However, shock sensitivity was not significantly different between the saline and phenylephrine groups (Supplementary Fig. 3E, shock sensitivity (a.u.), N = 15 in each group: saline group, 58,656 ± 2,240; phenylephrine group, 57,327 ± 2,492; p = 0.69). These results indicate that α1-AR stimulation in PFC can suppress locomotor activity and enhance fear expression. Next, a memory retrieval test was performed one day after the CFC in the absence of CNO. The freezing ratio of the mice administered phenylephrine was significantly increased compared with that of the saline group (Supplementary Fig. 3D, freezing ratio (%), N = 15 in each group: saline group, 15.0 ± 2.6; phenylephrine group, 31.8 ± 5.9; p = 0.015). To exclude the possibility that the phenylephrine infusion per se induced persistent fear, mice could explore the conditioning chamber without receiving an electrical shock following the phenylephrine or saline infusion. During the contextual exposure, the locomotor activity was significantly decreased in the phenylephrine group compared with the saline group (Supplementary Fig. 3 F, motility (a.u.): saline group, 75,111 ± 29,930, N = 8; phenylephrine group, 11,332 ± 5,014, N = 9; p = 0.04). In the retrieval test, the freezing behavior of the mice in the phenylephrine group was comparable with that of the mice in saline group (Supplementary Fig. 3 G; freezing ratio (%): saline group, 11.0 ± 4.5, N = 8; phenylephrine group, 13.6 ± 3.8, N = 9; p = 0.67). These results suggest that α1-AR stimulation in the PFC enhances fear expression, and augments fear memory formation.

## Discussion

In the present study, we used hM3Dq receptor to manipulate the neuronal activity in a subset of PFC neurons during the CFC. Freezing behavior during conditioning was augmented by stimulation of PFC neurons using hM3Dq. The activation of PFC neurons during conditioning resulted in the increase of freezing ratio in the conditioned context but not in the novel context. The c-fos imaging analysis showed that hM3Dq activation in PFC neurons did not change which neurons are reactivated during recent memory retrieval. Instead, more BLA neurons expressed c-fos protein following memory retrieval by modulation of PFC activity during conditioning. Therefore, our results show that PFC activation during conditioning augments fear expression and memory formation in the CFC.

The dorsomedial PFC regulate the expression of fear in rodents^10^. Inactivation of the PFC interferes with the expression of learned fear in the CFC^11^. Consistent with previous study, PFC activation by hM3Dq increased freezing behavior during shock-presenting period in CFC (Figs. 1E and 2C). Previous studies showed that inactivation of PFC do not change the freezing against to innate fear^11^ and activation of PFC projection to BLA decreases anxiety^18^. But in the present study, locomotor activity in the context before presenting shocks was significantly decreased by stimulating PFC neurons (Fig. 1D). It is not clear whether activity suppression relates to anxiety-like behavior. Further study is necessary to address this point. In the c-fos imaging analysis following conditioning, LH neurons were more activated by stimulating PFC neurons. PFC innervate the LH^32^. Stimulating LH terminals in the LC can enhance fear expression and consolidation in the auditory fear conditioning^33,34^. One possibility is that PFC activation enhances fear expression via the LH-LC pathway (Supplementary Fig. 2). The LC neurons are a major source of noradrenaline; they innervate the amygdala and the PFC. A recent study showed that the activation of noradrenergic neurons in the LC increased the expression of conditioned freezing behavior; the infusion of α2-AR (Gi-GPCR) agonist into the PFC blocked the fear expression induced by LC-activation^37^. In this study, we showed that the infusion of an α1-AR agonist into PFC during conditioning enhanced the freezing behavior during both the conditioning and retrieval phase (Supplementary Fig. 3 C and D). One possible explanation for this result is that the PFC-LH-LC loop circuit enhances fear induced by the US and increases fear memory formation (Supplementary Fig. 2). BLA has a reciprocal connection with the PFC and is innervated from the LC, while noradrenaline increases in the amygdala during fear conditioning, promoting fear memory consolidation^42,43^. In our experiment, the activation of PFC neurons during conditioning led to higher freezing behavior when mice were returned to the conditioned context but not in a new context, indicating that fear memory formation was enhanced (Fig. 1F,G). The c-fos analysis showed that more BLA neurons were activated following memory retrieval in the CNO group compared with the saline group (Fig. 5B). A previous study showed that silencing the synaptic terminals of PFC neurons in the BLA impairs memory retrieval^14^. Furthermore, an electrophysiological study showed that fear learning leads to the strengthening of PFC excitatory synapses in BLA principal neurons^44^. One possible explanation for this phenomenon is that fear-related circuits are activated by PFC neurons, which would strengthen the CS-US association in BLA, resulting in enhanced fear memory formation (Supplementary Fig. 2). However, we did not observe a significant increase in the activation of BLA neurons after PFC activation, although PFC innervates the BLA. This may be because the activation of BLA neurons through US input would mask modulation of PFC activation. More research is required to further describe the mechanism of memory enhancement caused by PFC activation.

In a previous study, hM3Dq was expressed in a random small population of BLA neurons. The excitation of these neurons during auditory fear conditioning enhanced memory formation, and pre-activated neurons were shown to be preferentially incorporated into a memory trace^26^. In this study, hM3Dq was expressed in a subset of PFC neurons and neuronal activity was increased by systemic administration of CNO. However, in the retrieval test, c-fos was not preferentially expressed in these pre-activated PFC neurons, in contrast to the results of the previous study reported above^26^. Engram cells for contextual fear memory in the PFC have been gradually developed^7,8^. Future studies are needed to analyze c-fos expression following remote memory retrieval. The immunohistochemical approach has limitation to test whether the cells activated by training are tagged and can be assessed again at retrieval. Therefore, to fully address the mechanism of memory allocation, additional approaches, such as *in vivo* imaging analysis, are necessary. In this study, we had hypothesized that the activation of PFC neurons during the conditioning phase would accelerate the consolidation process but could activate a random population of neurons, becoming non-selective noise for information processing and/or augmented aversive experience (e.g. electrical shock), which could be related to the increase in c-fos expression following conditioning. Unlike in the amygdala, where around 70% of neurons are activated by a tone or electrical shock^45,46^, the manipulation of a specific neuronal circuit is necessary to address the cellular mechanisms of memory allocation in the PFC, such the synaptic connection with BLA and LC.

In summary, the findings presented here show that excitation of PFC neurons during conditioning enhances fear expression and memory formation in CFC.

## Materials and Methods

### Animals

All experimental protocols were evaluated and approved by the Regulation for Animal Research at Tokyo University of Science. All experiments were conducted in accordance with the Regulations for Animal Research at the Tokyo University Science. Mice were individually housed in a 12-hour (7:30 am to 7:30 pm) light/dark cycle with food and water ad libitum. We used 3 to 4 month-old C57BL/6 J male mice (SLC Japan).

### Virus vectors

The pAAV-EF1a-DIO-hM3D(Gq)-mCherry plasmid was purchased from Addgene. Then, the AAV was produced as described previously^47^. The titer of AAV_dj/8_ EF1a-DIO-hM3D(Gq)-mCherry titrations was 5.1 × 10^11^ genome copy per mL. The AAV_8_-CaMKIIa-HA-hM3D(Gq)-IRES-mCtrine (3.1 × 10^12^ genome copy/mL) and AAV_8_-CaMKIIa-GFP-Cre (4.4 × 10^12^ genome copy/mL) were purchased from the Vector Core at the University of North Carolina at Chapel Hill.

### Stereotactic surgery and cannula placement

The mice were anesthetized with pentobarbital (80 mg/kg of body weight by intraperitoneal injection), given carprofen (5 mg/kg of body weight; subcutaneous injection), and the fully anesthetized mice were placed in a stereotactic apparatus (Narishige, Japan). A 2-mm diameter craniotomy was performed above the PFC. A 200 nL virus solution was bilaterally infused into the dorsomedial PFC using a Hamilton syringe through a glass micropipette at the following coordinates: relative to bregma (mm): anteroposterior axis (AP): +1.8, mediolateral axis (ML): ±0.2, and dorsoventral axis (DV): −1.8 from dura mater, taken from the mouse brain atlas^31^ at a rate of 0.1 μL/min. A glass capillary was left in place for an additional 5 min. A guide cannula that was composed of two stainless steel pipes (internal diameter, 0.30 mm; outer diameter, 0.46 mm; center to center distance, 0.5 mm, Plastics One, Roanoke, VA, USA) was implanted above the PFC areas (AP, +1.8 mm; ML, ±0.25 mm; DV, 0.0 mm from the dura mater), and a dummy cannula was inserted into the guide cannula (1.5-mm projection from the bottom of a guide cannula). The injection cannula was inserted into the guide cannula which targeted the PFC (AP, +1.8 mm; ML, ±0.25 mm; DV, 1.8 mm from the dura mater). The guide cannula and micro-screws were fixed on the skull with dental cement. Behavioral tests were implemented around 4 weeks for the experiment using AAV and 2 weeks for other pharmacological experiments after surgery to allow for sufficient expression of genes and recovery of mice.

### Immunohistochemistry and analysis of c-fos positive neurons

Mice were deeply anesthetized with pentobarbital, given carprofen and transcardially perfused with 4% (w/v) paraformaldehyde in 0.1 M sodium phosphate buffer, pH 7.4. The brains were excised, postfixed with the same fixative at 4 °C overnight and equilibrated in 30% (w/v) sucrose in phosphate-buffered saline (PBS) as a cryoprotectant. The brains were embedded in OCT compound (Sakura Finetech), and frozen coronal sections (50 μm) were prepared. Free-floating sections were incubated with 0.2% (v/v) Triton X-100 in PBS, and then blocked with 5% (v/v) goat serum and 0.2% (v/v) Triton X-100 in PBS. Sections were incubated with primary antibodies against c-fos (1:200; Synaptic Systems; 226 003), GFP (1:1000; Aves Labs Inc.; GFP-1020) and mCherry (1:5000; Thermo Fisher Scientific; M11217) at 4 °C for two nights. Then, sections were incubated with an Alexa546- or Alexa647-conjugated anti-rabbit IgG, Alexa546-conjugated anti-rat IgG, and Alexa488-conjugated anti-chicken IgY (1:300; Life Technologies). Nuclei were stained using DAPI (Life Technologies). Sections were imaged with a confocal microscope (Olympus; FV1000). Confocal images of the PFC, BLA, LH, NAc, CA3 and DG were acquired using a 20× objective lens.

Manual cell counts were performed by an experimenter blinded to the treatment. Small, bright uniformly DAPI-stained nuclei from the putative glial cells were not counted. Each brain area was identified according to a mouse brain atlas^31^ (Supplementary Fig. 4; analyzed positions: PFC, AP, from around +2.1 to +1.5 mm; NAc, AP from around +1.7 to +1.3 mm; LH, AP from around −1.0 to −1.4 mm; BLA, AP from around −1.6 to −2.0 mm; CA3 and DG, AP from around −1.8 to −2.2 mm). Two to four slices were analyzed for each mouse. Neighbor slices were separated by a distance of 0.2 mm. The probability of c-fos+ on hM3Dq+ or DAPI + neurons was calculated for each mouse studied.

### CFC test

Mice were housed individually after surgery. All training and testing experiments were performed during the light cycle. A fear-conditioning shock chamber consisting of a Plexiglass front, gray sides, and back walls (18 W × 17 D × 40 H cm) with 26 stainless steel grids on the floor (Muromachi) was used. Each mouse was placed into the conditioning chamber and allowed to explore for 3 min (pre-shock period). Then, mice received three shocks (0.4 mA for 2 s), with a 60-s interstimulus interval in between for hM3Dq experiments and a single shock for phenylephrine experiments. The mice were left in the conditioning chamber after the last shock for 1 min. For the memory retrieval test, mice were returned to the same conditioning chamber 24 hours following CFC, and their behavior was monitored for 5 min. For the memory retrieval test in a novel context, mice were allowed to explore for 3 min followed by three shocks in the same conditions as described above. The following day, mice were returned to the chamber consisting of a triangular prism, white walls (one side is 18 ×40 H cm) with a white paper towel on the floor. The freezing behavior and motility of mice were monitored using a video camera and analyzed using DVTrack video tracking system (Muromachi). Freezing was defined as the complete absence of movement, except for those related to respiration. The duration of the freezing behavior was timed when the mouse showed sustained freezing behavior for more than 2 s. Motility was calculated as the cumulative area of movement (in pixels) per 0.1 s. The mean locomotor activity was calculated as the averaged motility per minute during the pre-shock period. Shock sensitivity was calculated as the motility for 2 s giving an electrical shock.

To activate hM3Dq receptor at the time of learning, saline or 3 mg/kg of CNO (Enzo Life Sciences) dissolved in saline was intraperitoneally injected to the mice 30 min before the CFC. For the immunohistochemical analysis of c-fos protein, brains were extracted 90 min after the CFC or retrieval test.

### Drug infusion

(R)-(-)-phenylephrine hydrochloride (Wako; 61–76–7) was used for the α1-AR agonist, respectively. Ten minutes prior to the CFC, mice received infusions of 0.5 μL of phenylephrine (10 mg/mL in saline) at a rate of 0.2 μL/min. The injection cannula was left in place for 2 min following the infusion.

### Statistical analyses

For comparisons of the two groups, we used unpaired two-tailed t-tests. Differences were considered to be statistically significant when the probability value was <0.05. The p-value and effect size (Cohen’s d) are listed in Supplemental Table 1.

## Supplementary information

Supplementary Figure. 1, Supplementary Figure. 2, Supplementary Figure. 3, Supplementary Figure. 4, Supplementary Table 1.

## Data Availability

The datasets generated and analyzed in the current study are available from the corresponding author on reasonable request.
