# Supplementary material for: Excitation of prefrontal cortical neurons during conditioning enhances fear memory formation
Source: Sci Rep. 2020 May 25;10:8613. doi: 10.1038/s41598-020-65597-7 (PMC7248099; doi:10.1038/s41598-020-65597-7)
Supplement: Supplementary file 1 — Supplementary Figure. 1, Supplementary Figure. 2, Supplementary Figure. 3, Supplementary Figure. 4, Supplementary Table 1. [file 41598_2020_65597_MOESM1_ESM.pdf]

Supplementary Information

Excitation of prefrontal cortical neurons during conditioning enhances fear memory formation

Natsumi Shibano, Mio Yamazaki, Tomoki Arima, Konami Abe, Marin Kuroda, Yuki Kobayashi, Shigeyoshi Itohara, Teiichi Furuichi and Yoshitake Sano.

Supplementary Figure. 1 Expression of hM3Dq in PFC

Representative pictures showing hM3Dq in each mouse. The CaMKIIa-hM3Dq-IRES-mCitrine AAV was infused into PFC. Coronal sections at intervals of 200 μm are shown. The scale bar indicates 1000 μm in (A) and 100 μm in (B).

Supplementary Figure. 2 Proposed model for fear expression and memory formation by activating PFC

PFC; dorsomedial prefrontal cortex, BLA; basolateral amygdala, HPC; hippocampus, LH; lateral hypothalamus, LC; locus coeruleus.

Supplementary Figure. 3 α1 adrenergic receptor activation on PFC enhances fear expression and memory formation. (A) The left panel shows the experimental schema. Phenylephrine (Phe) was bilaterally infused into PFC via infusion cannula before conditioning. The right representative picture shows the damaged area by cannula, which is depicted by white dot lines. (B) Mean locomotor activity for 3 min before receiving an electrical shock during conditioning. (C) Mean freezing ratio for 1 min during the shock-presenting period. (D) Mean freezing ratio for 5 min during the retrieval test. (E) Shock sensitivity. (B-E; N = 15 animals per group). (F) Mean locomotor activity for 3 min in the

conditioned context. (G) Mean freezing ratio for 5 min during the retrieval test. In this experiment, an electrical shock was not delivered to mice during conditioning. (F and G; saline group, n = 8; Phe group, n = 9 animals). Data are represented as means ± SEM; $*p < 0.05$ and $**p < 0.01$. (Saline-infused mice, black columns; Phe-infused mice, green columns).

Supplementary Figure. 4 References of brain areas for c-fos analysis.

Schema of coronal sections at each AP position (AP = +1.9 mm; PrL, AP = +1.7 mm; NAc, AP = −1.2 mm; LH, AP = −1.8 mm; BLA, CA3 and DG). Representative analyzed areas were depicted by a red line.

Supplementary Table 1 List of the p-value and effect size.

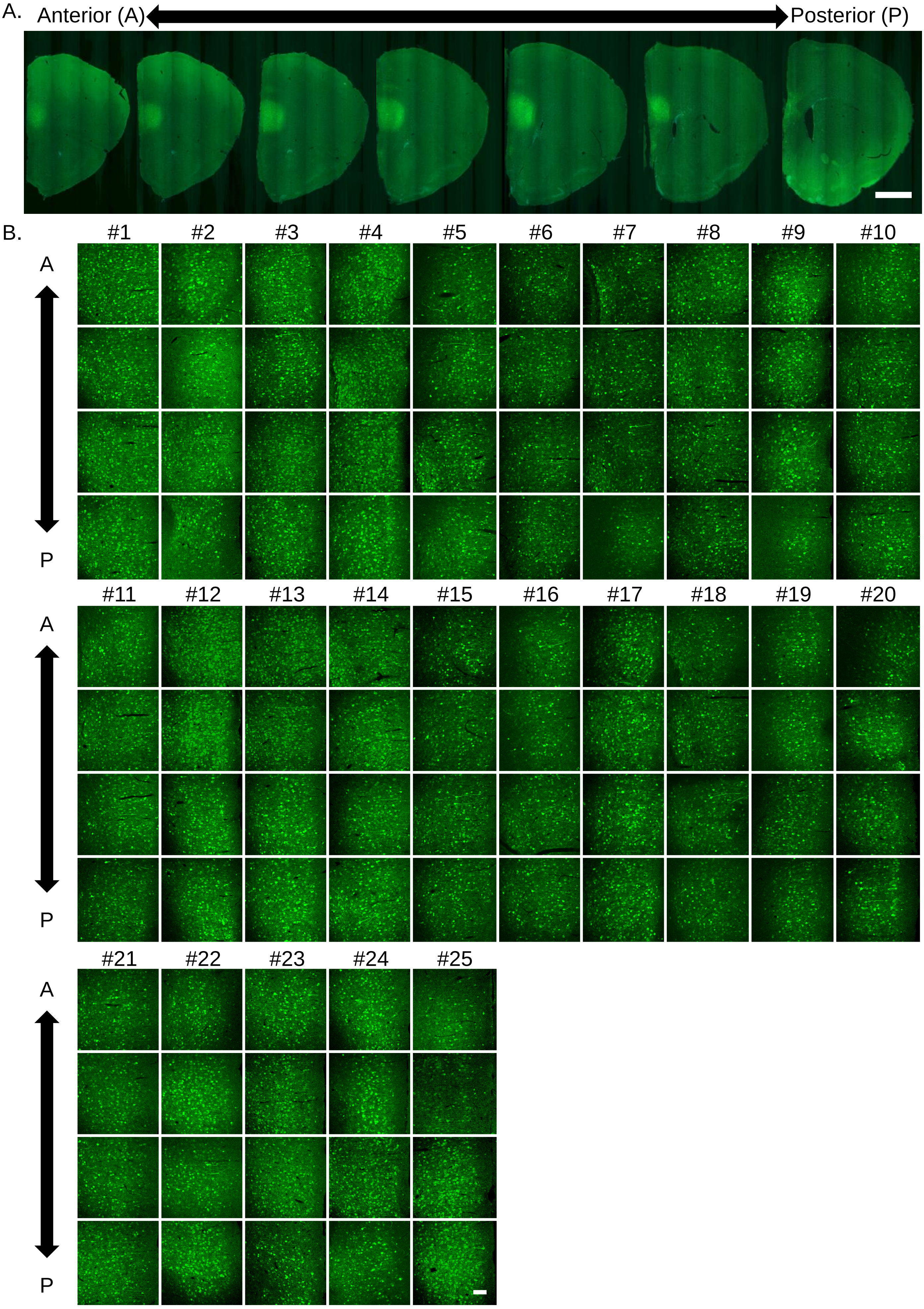

Supplementary Figure. 1

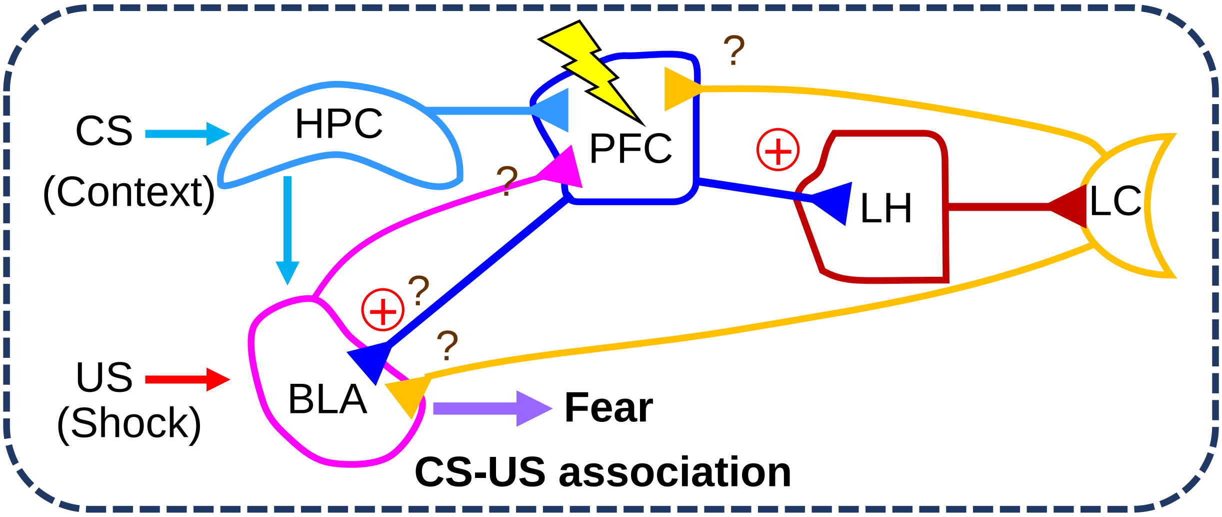

Supplementary Figure. 2

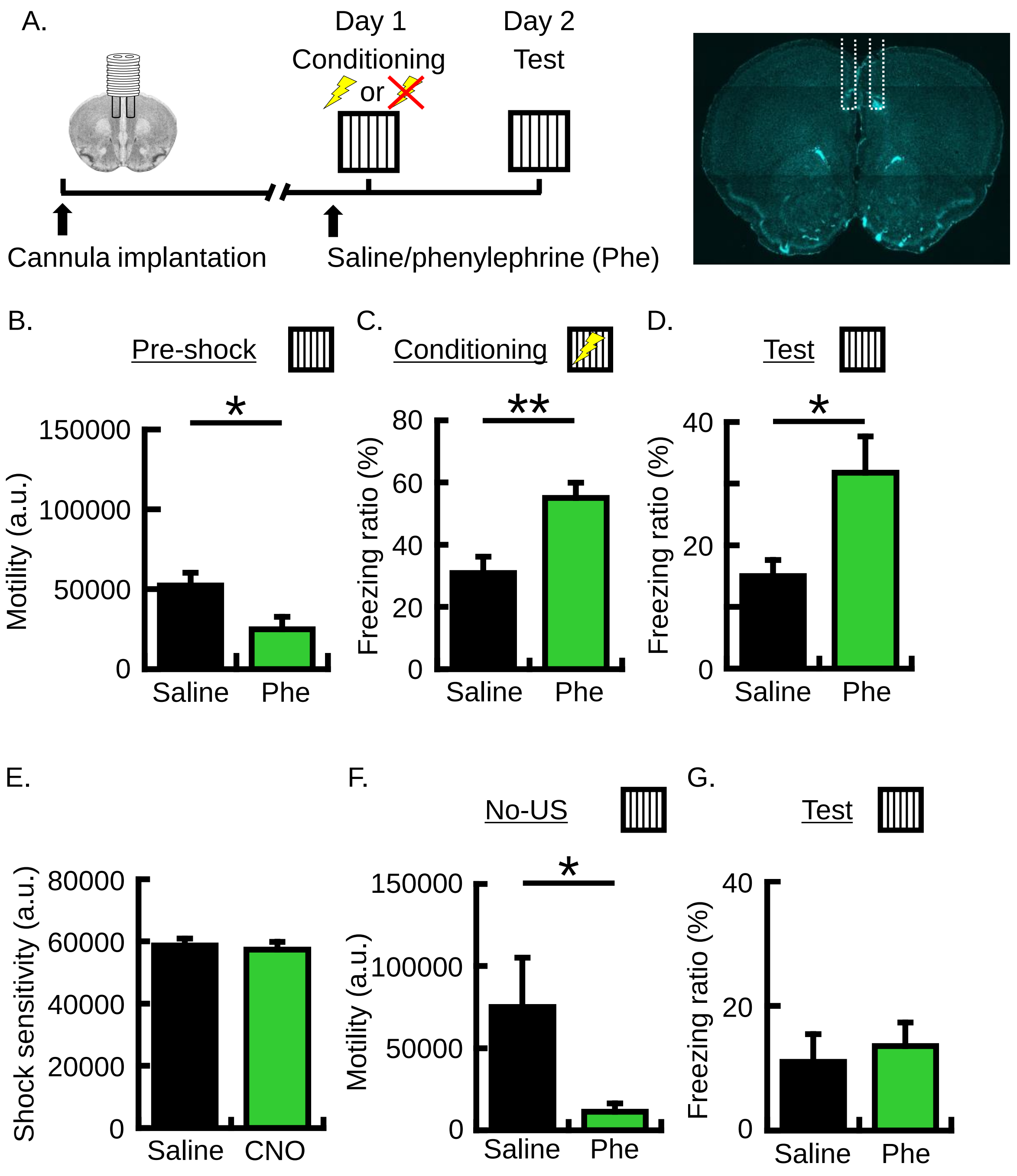

Supplementary Figure. 3

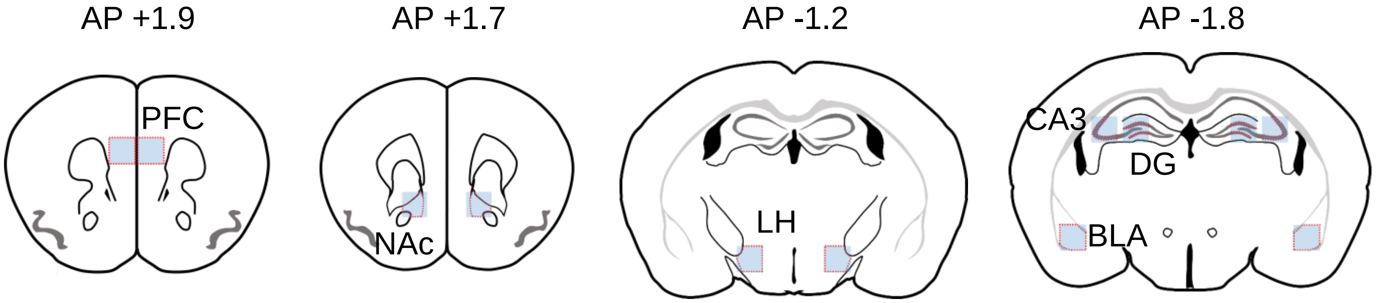

Supplementary Fig. 4

Fig. #	Contents	p values	# of samples	effect size d
1-C	c-fos+/hM3+ neurons (%)	0.0003	4:4	6.23
1-D	Motility (a.u.)	0.0006	13:12	1.66
1-E	Freezing ratio (%)	0.005	13:12	1.31
1-F	Freezing ratio (%)	0.032	13:12	0.95
2-C	Freezing ratio (%)	0.004	17:17	1.11
3-A	c-fos+/DAPI (%)	0.005	6:6	2.27
3-D	c-fos+/DAPI (%)	0.030	6:6	1.60
5-B	c-fos+/DAPI (%)	0.030	6:6	1.60
S3-B	Motility (a.u.)	0.021	15:15	0.92
S3-C	Freezing ratio (%)	0.002	15:15	1.39
S3-D	Freezing ratio (%)	0.015	15:15	0.98
S3-F	Motility (a.u.)	0.041	8:9	1.15

Supplementary Table. 1
